# Rare Presentation of Pityriasis Lichenoides et Varioliformis Acuta: A Case of Mucosal and Face Involvement in a 15‐Year‐Old Male

**DOI:** 10.1002/ccr3.71010

**Published:** 2025-09-26

**Authors:** Sandesh Shah, Joshana Shrestha, Smriti Piya

**Affiliations:** ^1^ Department of Dermatology Nepal Medical College and Teaching Hospital Kathmandu Nepal

**Keywords:** dermoscopy, pityriasis lichenoides chronica, PLEVA, varioliform scar

## Abstract

Pityriasis lichenoides et varioliformis acuta (PLEVA) is a rare inflammatory skin disorder, and mucosal involvement is exceptionally uncommon. We present a 15‐year‐old male who developed PLEVA with facial and mucosal involvement, preceded by fever. Dermoscopy revealed dotted and linear vessels with micaceous scales. Histopathology showed focal parakeratosis, epidermal exocytosis of neutrophils and lymphocytes, and dense inflammatory infiltrates extending into the subcutaneous tissue. The patient responded well to doxycycline, achieving complete resolution, though varioliform scarring and hyperpigmentation remained. This case underscores the rarity of mucosal involvement in PLEVA and highlights the importance of early recognition and treatment to minimize long‐term cutaneous damage.

## Graphical abstract

Graphical abstract illustrating a rare case of PLEVA with mucosal and facial involvement in a 15‐year‐old male, successfully treated with doxycycline.

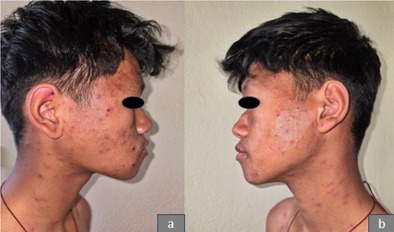

**Summary**

- Pityriasis lichenoides et varioliformis acuta (PLEVA) can present with atypical features, including mucosal involvement and facial lesions. Early recognition and prompt treatment, even with antibiotics like doxycycline, may prevent complications such as scarring. Clinicians should consider PLEVA in the differential diagnosis of acute vesiculopapular eruptions with systemic symptoms.

## 1 Introduction

Pityriasis lichenoides et varioliformis acuta (PLEVA), also known as Mucha‐Habermann disease, is a rare T‐cell lymphoproliferative disorder that can occur at any age but most commonly affects children and young adults, with a slight male preponderance [1]. The pathogenesis of PLEVA is thought to involve two mechanisms: a T‐cell dyscrasia and an aberrant immune response to viral, bacterial, or protozoal infections [2].

PLEVA is marked by the sudden appearance of asymptomatic multiple small erythematous papules that rapidly progress into polymorphic lesions with varying forms, including vesicles, pustules, hemorrhagic crusted papules, and shallow ulcers. These lesions typically resolve within weeks to months but can occasionally leave behind long‐lasting complications such as hyperpigmented or hypopigmented, varioliform scars. The lesions primarily affect the anterior trunk, flexural areas, and proximal extremities, though they can also occur in other parts of the body, sparing the mucosa [1, 3].

PLEVA typically has a self‐limiting course, with lesions resolving spontaneously over time. However, in certain cases, the condition may persist and follow a more recalcitrant trajectory, characterized by recurring flare‐ups and varying lengths of remission between episodes [3].

To our knowledge, this is the first documented case of PLEVA presenting with significant facial and mucosal involvement in a 15‐year‐old male from Nepal. This case highlights the atypical nature of the condition and underscores the rarity of such presentations.

## 2 Case History/Examination

A 15‐year‐old male presented to the dermatology department with multiple non‐pruritic reddish‐brown elevated lesions, symmetrically distributed across the face, trunk, upper and lower limbs, and oral cavity for 10 days. The patient reported a history of fever (38.3°C) prior to the appearance of the lesions; however, no other systemic symptoms were present at the time.

**FIGURE 1:**
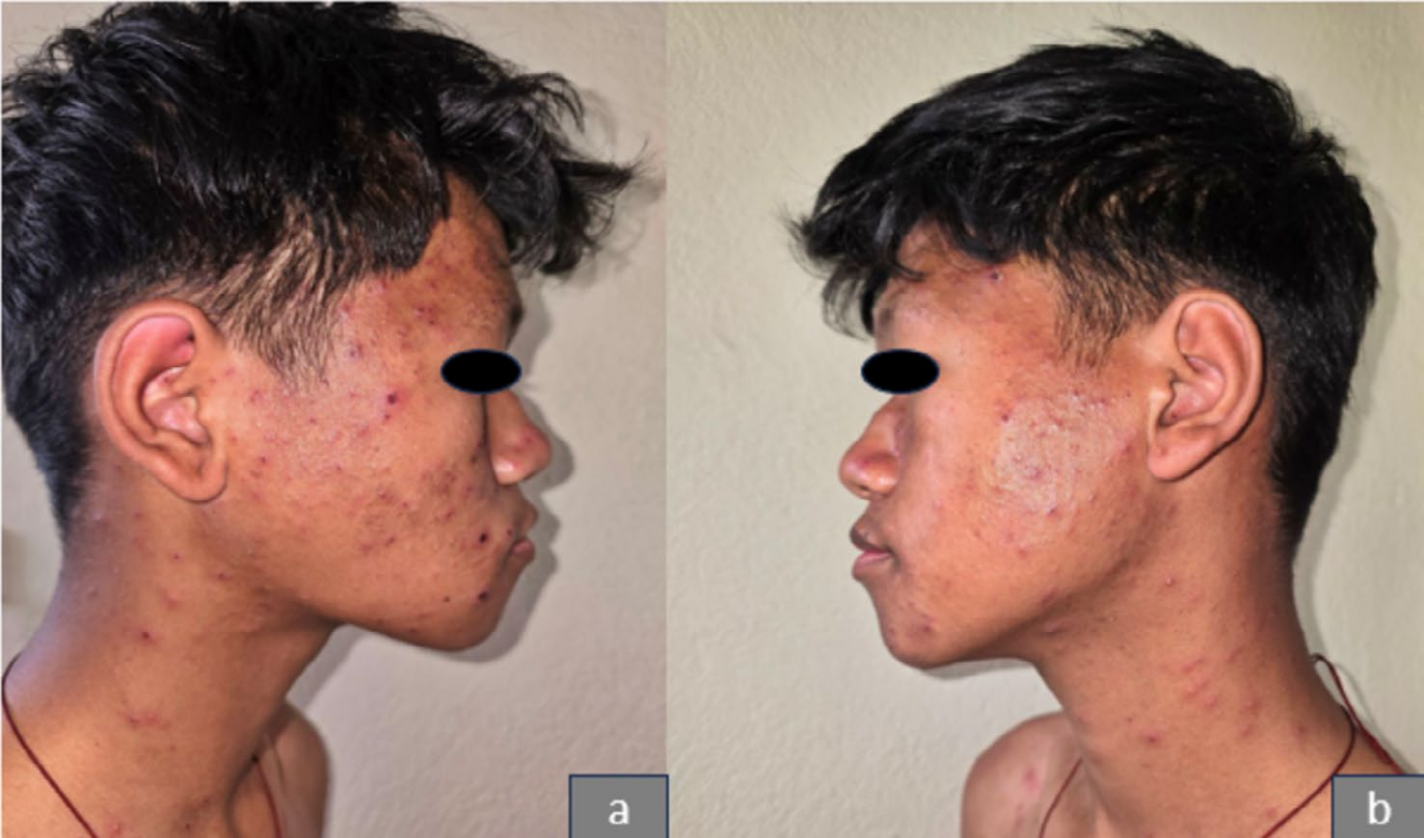
Showing erythematous papules, pustules, and crusted lesions over the face and neck.

**FIGURE 2:**
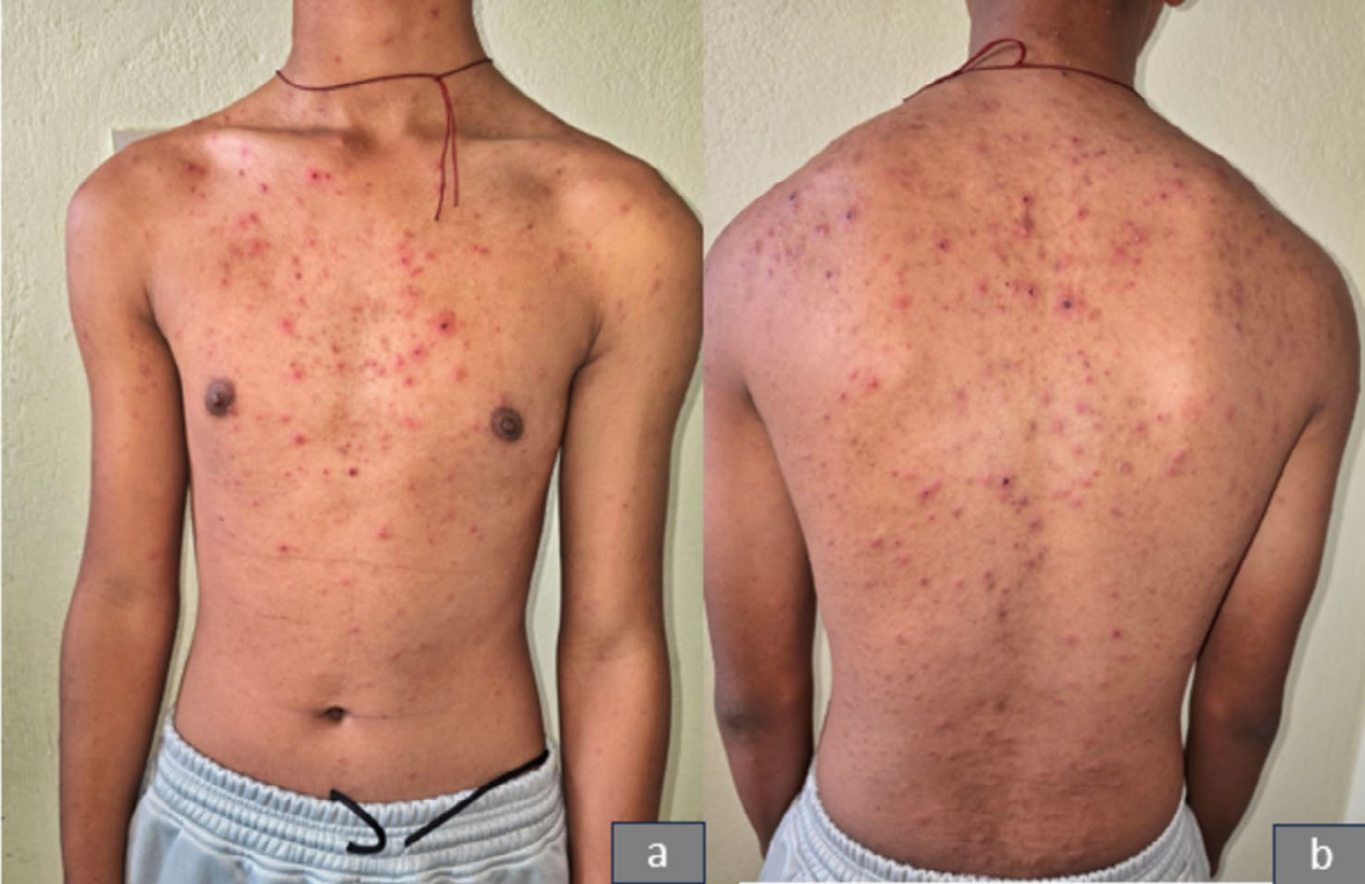
Showing polymorphic lesions, including papules, pustules, and crusted lesions over the trunk.

**FIGURE 3:**
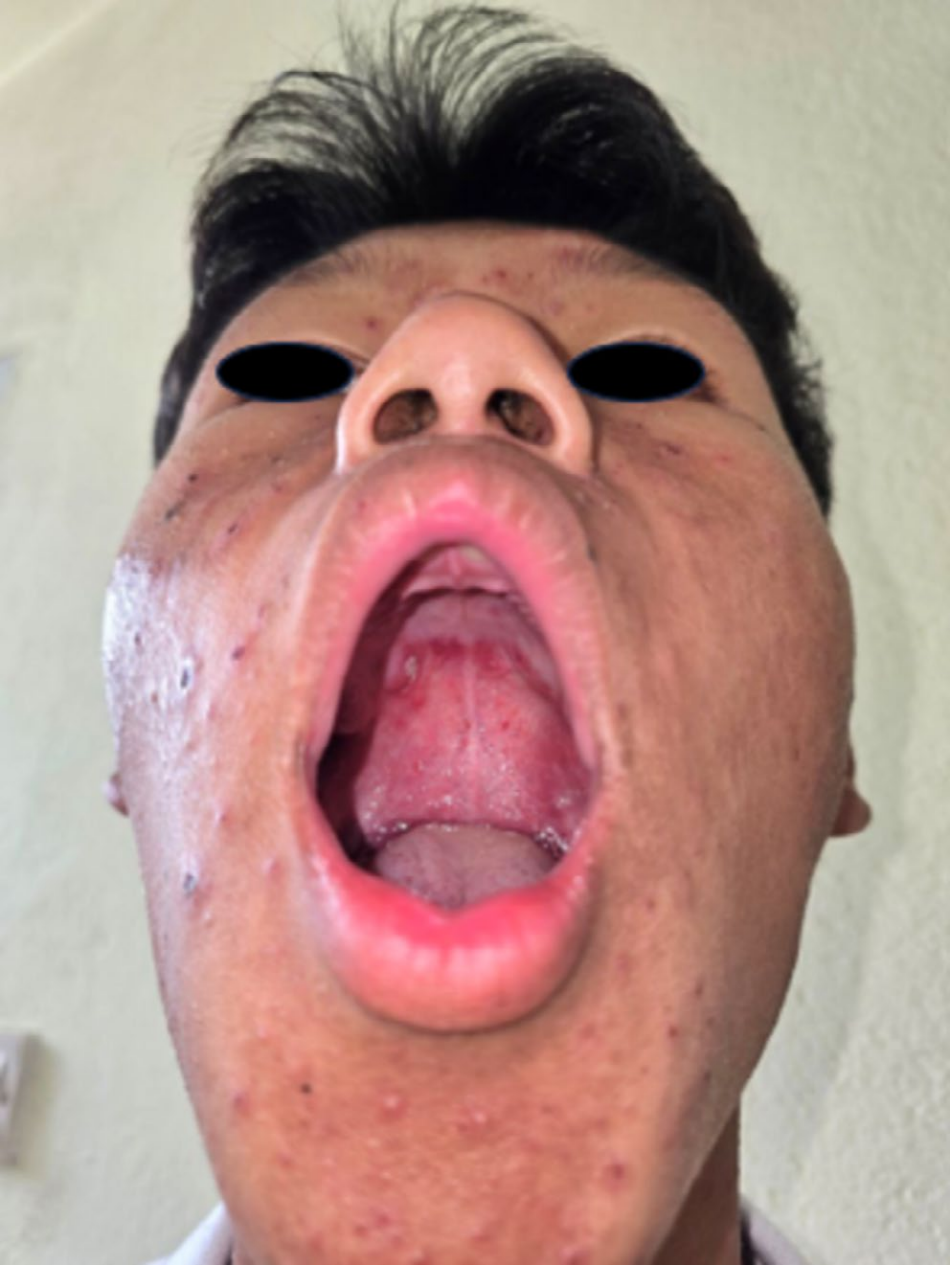
Showing involvement of the hard palate.

On clinical examination, erythematous papules of varying sizes, ranging from 3 to 15 mm, were observed in different stages of evolution. These lesions were spread over the face (Figure 1), trunk (Figure 2), and extremities, including the involvement of the hard palate (Figure 3). Some lesions showed a vesicular and pustular center, while others exhibited a micaceous scale at the center, with some developing different stages of healing (Figures 1 and 2). Additionally, erosions were noted in certain areas of the lesions, further highlighting the polymorphic nature of the eruption.

**FIGURE 4:**
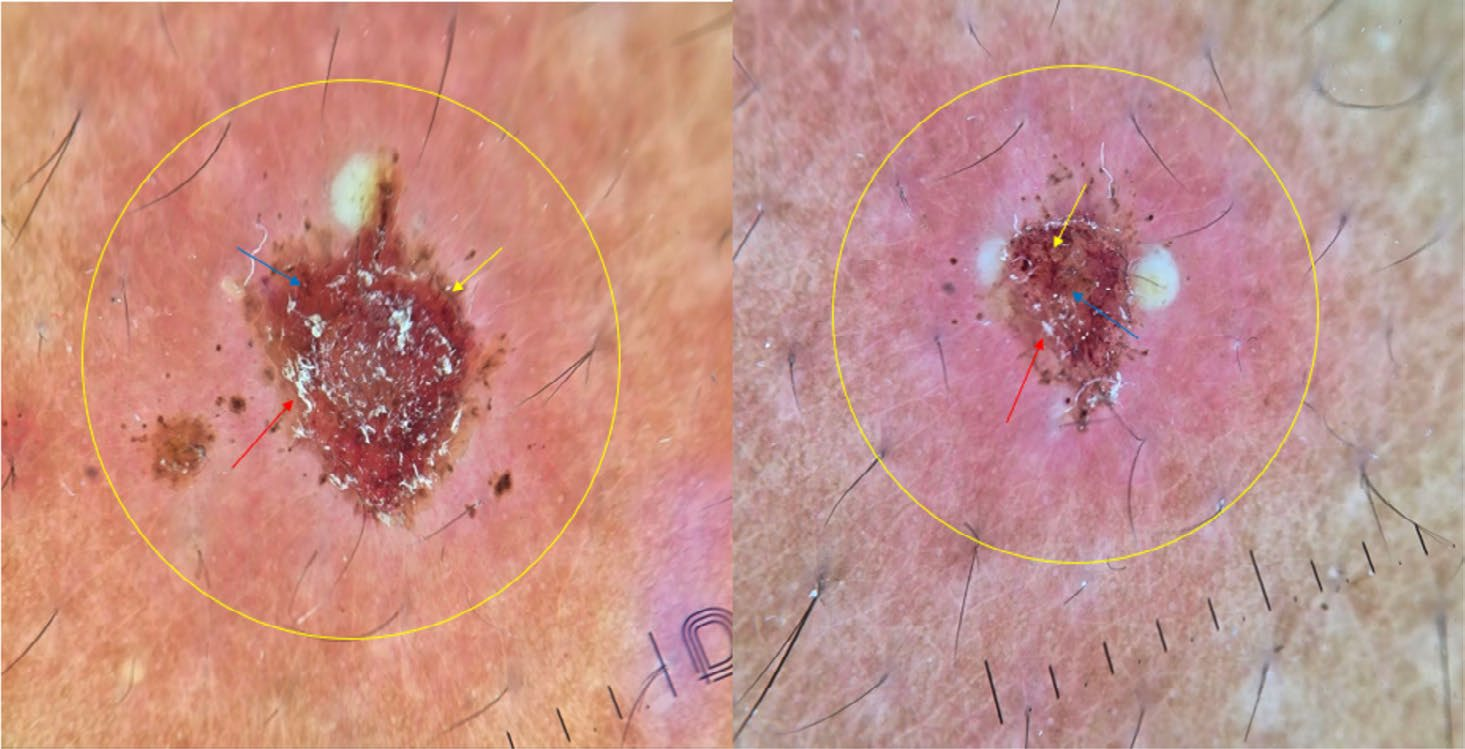
Dermoscopy showing dotted vessels (blue arrow), irregular linear vessels (yellow arrow), white scales (red arrow), erythematous background (yellow circle).

Dermoscopic examination revealed a range of vascular patterns, including dotted vessels and linear‐irregular vessels. The vascular arrangement was predominantly peripheral in most lesions. Scales were present in some of the lesions, with most appearing centrally located, while others exhibited a ring‐like arrangement around the lesion. Additionally, the background of nearly all the lesions appeared erythematous or pink (Figure 4). These dermoscopic findings provide further insight into the polymorphic nature of the condition and its inflammatory characteristics.

## 3 Investigations and Treatment

A Tzanck smear from the base of the intact vesicle was performed, and no multinucleated giant cells were found. Laboratory findings revealed a normal complete blood count and renal and liver function; C‐reactive protein and erythrocyte sedimentation rate were elevated. Serology screening, including human immunodeficiency virus (HIV), hepatitis C virus (HCV), hepatitis B surface antigen (HBsAg), and Venereal Disease Research Laboratory (VDRL), was negative. Antinuclear antibody was negative.

**FIGURE 5:**
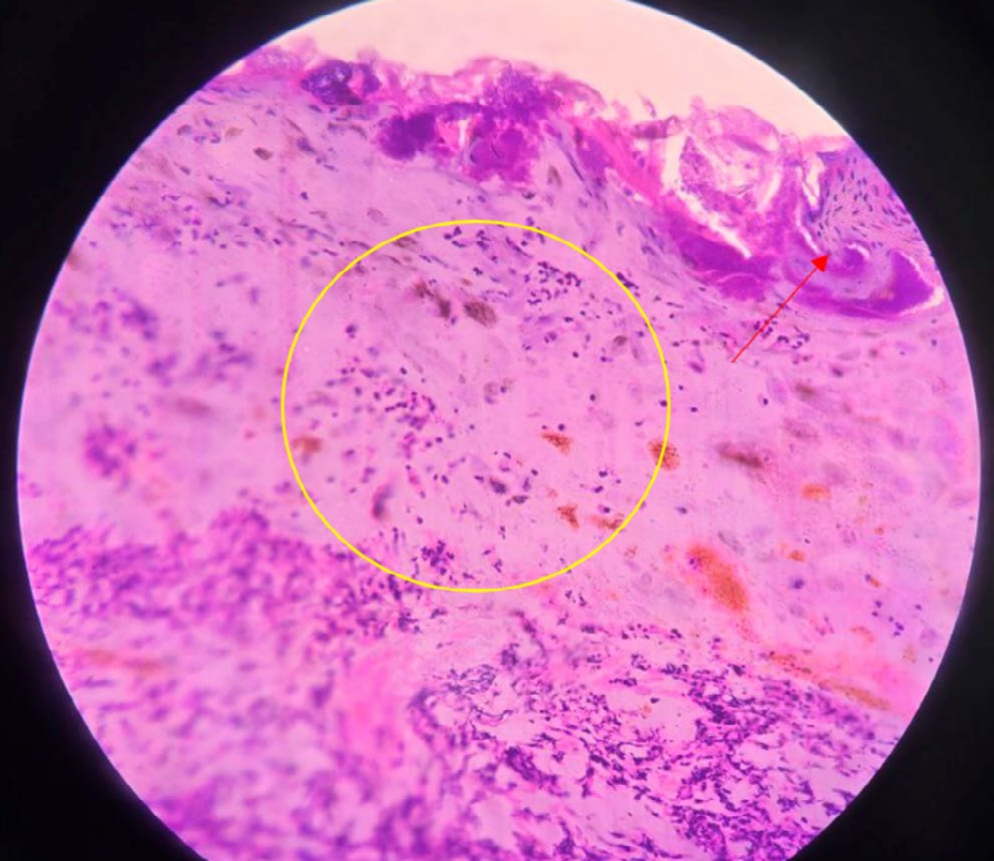
Hematoxylin and eosin stain at 40× showing focal parakeratosis (red arrow), exocytosis of the neutrophils and lymphocytes in the epidermis (yellow circle).

**FIGURE 6:**
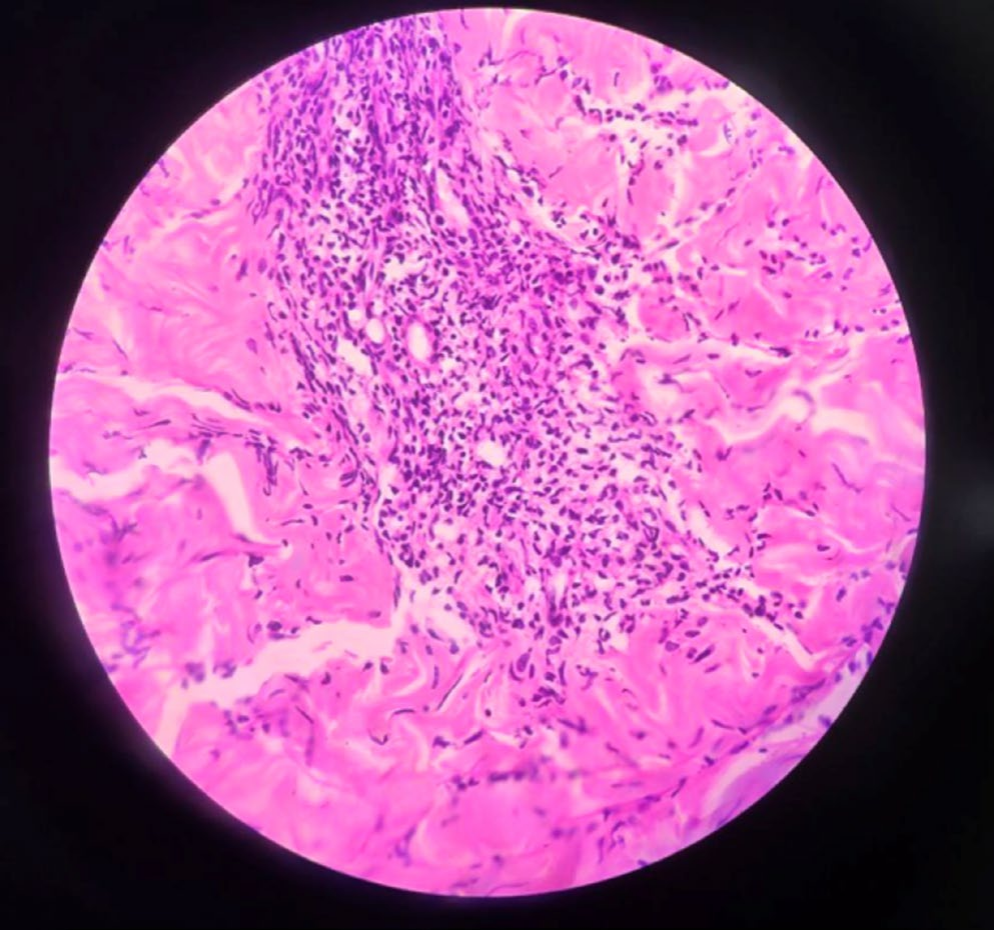
Hematoxylin and eosin stain at 40× showing perivascular mixed inflammatory infiltrates.

Histopathological examination revealed focal parakeratosis and exocytosis of neutrophils and lymphocytes (Figure 5). The dermis showed dense sheets of mixed inflammatory infiltrates, including neutrophils, lymphocytes, macrophages, and plasma cells, extending into the subcutaneous tissue. Perivascular mixed inflammatory infiltrates were also observed (Figure 6). Immunohistochemistry could not be performed due to the unavailability of the test and financial constraints.

The clinical, dermoscopic, and histological findings were suggestive of pityriasis lichenoides et varioliformis acuta (PLEVA). The patient was treated with doxycycline 100 mg twice daily for 3 weeks, along with chlorhexidine mouthwash and supportive care, including the use of emollients.

## 4 Outcome and Follow‐Up

**FIGURE 7:**
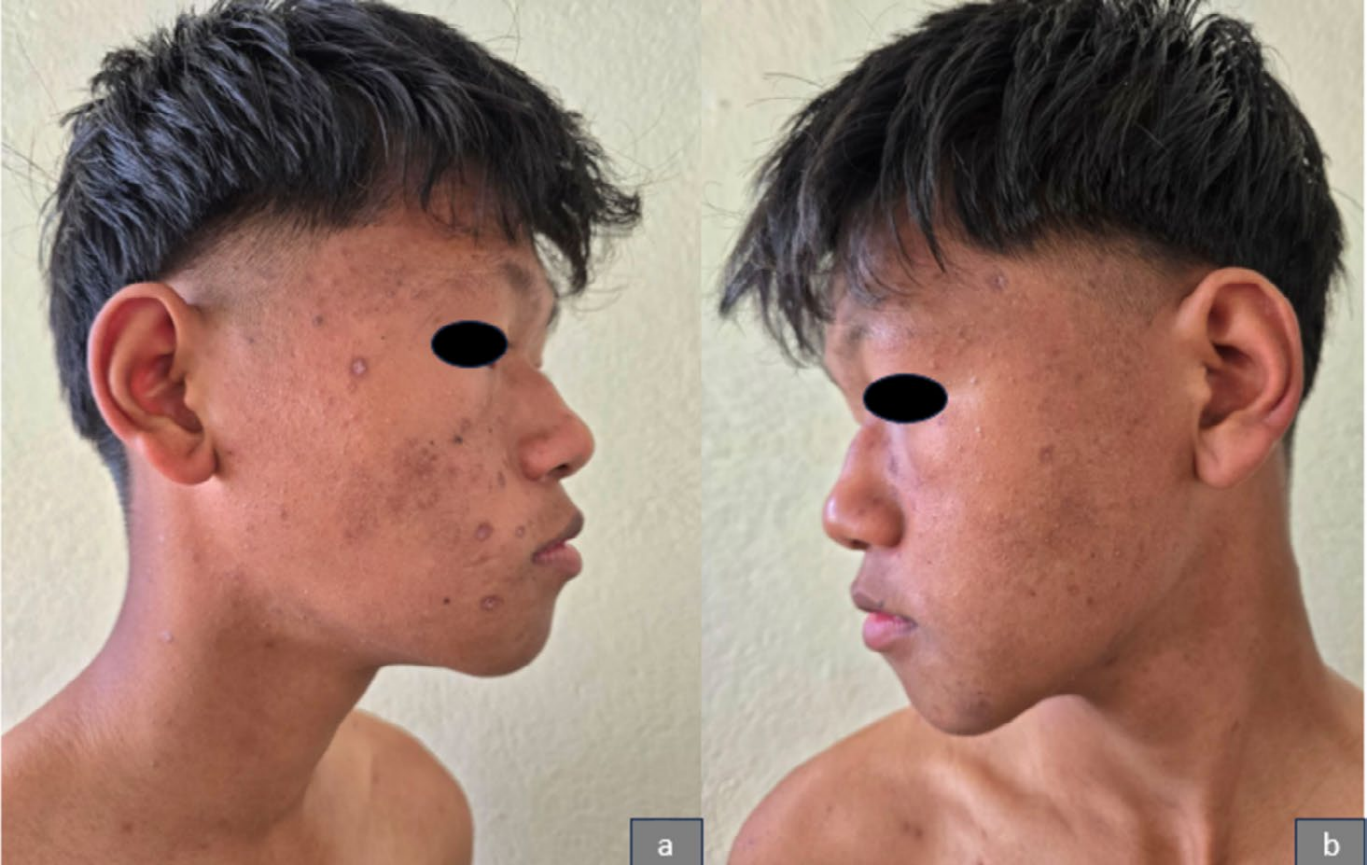
Showing varioliform scars and residual hyperpigmentation over the face after 3 weeks posttreatment.

**FIGURE 8:**
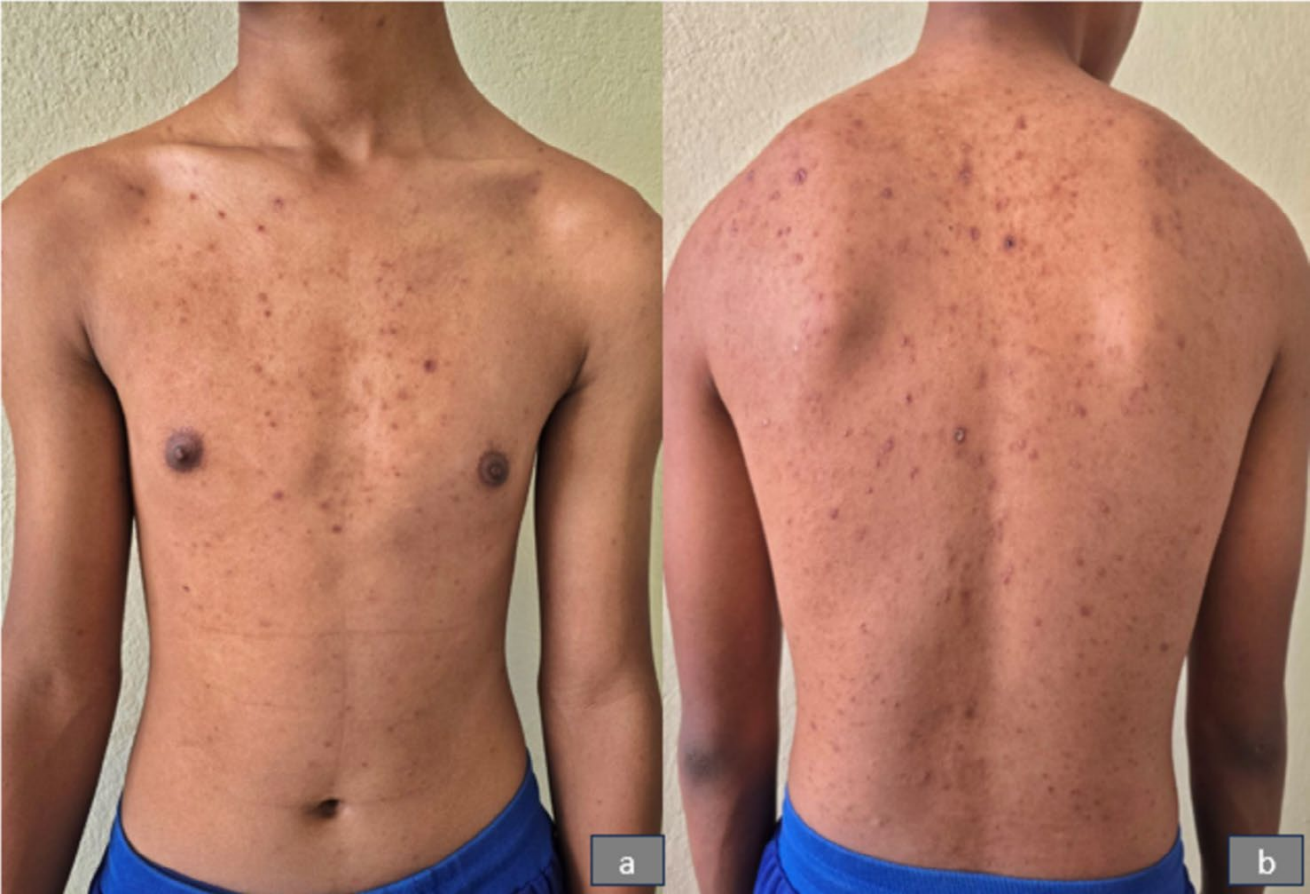
Showing varioliform scars and residual hyperpigmentation over the trunk after 3 weeks posttreatment.

The patient was followed up after 3 weeks, showing varioliform scars on the face (Figure 7) and trunk (Figure 8) along with residual hyperpigmented areas. The patient is on topical steroids and is being followed up monthly to monitor for any signs of relapse or recurrence.

## 5 Discussion

Pityriasis lichenoides et varioliformis acuta (PLEVA) is a rare inflammatory skin disorder that belongs to the broader spectrum of pityriasis lichenoides, which also includes pityriasis lichenoides chronica (PLC) and febrile ulceronecrotic Mucha‐Habermann disease (FUMHD) [3].

The exact etiology of PLEVA remains unclear, but several infectious agents have been implicated, including HIV, Varicella‐Zoster virus, Epstein–Barr virus, Cytomegalovirus, Parvovirus B19, Adenovirus, *Staphylococcus*, *Streptococcus*, *Mycoplasma*, and *Toxoplasma*. Additionally, certain vaccinations (such as measles, mumps, rubella (MMR), and tetanus) and various medications, including chemotherapeutic agents, antihistamines, hormones, and herbal treatments, have been associated with its onset [3].

Recent studies suggest a multifactorial pathogenesis involving infectious triggers and immunological dysregulation. A type IV hypersensitivity reaction, mediated by cytotoxic T‐cells, may play a central role, leading to keratinocyte apoptosis and vascular injury. Additionally, an immune complex‐mediated mechanism has been proposed. Another widely accepted theory is that pityriasis lichenoides represents a T‐cell lymphoproliferative disorder, with evidence of monoclonal T‐cell populations in affected tissues [2]. PLEVA shares clinical and histopathological similarities with lymphomatoid papulosis and large plaque psoriasis, and in rare cases, it has been reported to progress to cutaneous lymphoma, supporting the theory of atypical lymphocytic proliferation in its development [3, 4, 5, 6].

In our case, a 15‐year‐old male presented with PLEVA involving the face and mucous membranes, preceded by a fever. This presentation is considered uncommon, as PLEVA typically affects the trunk and extremities, with mucosal involvement being rare. A case report by Pereira et al. described a 63‐year‐old male with polymorphous lesions on the trunk and extremities, particularly in flexural areas, though mucosal involvement was not observed [7]. Similarly, Ediale et al. reported a 62‐year‐old woman with lesions on the palms, forearms, and dorsal feet, but without any facial or mucosal involvement [8]. In another case, a 3‐year‐old male presented with generalized eruptions, sparing the face and mucous membranes, further emphasizing the rarity of such involvement in PLEVA [9].

However, PLEVA can sometimes present with atypical features. For instance, a 27‐year‐old male developed extensive hemorrhagic vesiculopustules involving the trunk, extremities, face, oral mucosa, and genital region [10]. This case highlights that, although uncommon, PLEVA can affect facial and mucosal areas, expanding the recognized clinical spectrum of the disease.

Systemic symptoms, such as fever, occurring before the onset of PLEVA lesions have been documented in several cases. For instance, a case report described a 6‐year‐old boy who developed PLEVA following a measles‐rubella vaccination; however, he did not experience fever or other systemic manifestations. In contrast, our patient reported a fever of 38.3°C prior to the appearance of skin lesions, consistent with other studies where febrile episodes preceded the cutaneous manifestations of PLEVA [11].

The presence of fever before lesion onset in our case aligns with the hypothesis that PLEVA may be triggered by infectious agents or represent an immune‐mediated response. Recognizing such atypical presentations is essential for ensuring accurate diagnosis and appropriate management of the condition.

PLEVA is characterized by unique dermoscopic features that have been described in several studies. A study investigating dermoscopic findings in different inflammatory dermatoses found that dotted vessels were the most frequently observed vascular pattern, present in 70% of cases. Although this study was not specific to PLEVA, the high occurrence of dotted vessels aligns with our observations and findings from another PLEVA‐focused research [12]. In our case, the combination of dotted and linear‐irregular vessels with peripheral arrangement and central scaling supports a dermoscopic pattern indicative of vascular inflammation and epidermal disruption, which aligns with existing descriptions in inflammatory papulosquamous disorders. Dermoscopy serves as a valuable noninvasive adjunct to clinical examination, particularly in atypical or evolving cases of PLEVA.

The histopathological features of PLEVA are marked by a dense, band‐like lymphocytic infiltrate in the dermis, along with vacuolar degeneration of the basal layer, significant exocytosis, and erythrocyte extravasation, sometimes accompanied by epidermal necrosis. These characteristics distinguish PLEVA from other conditions, such as pityriasis rosea and subacute eczematous dermatitis [13].

The histopathological findings in our case align with these descriptions, showing focal parakeratosis, exocytosis of neutrophils and lymphocytes, dense mixed inflammatory infiltrates extending into the subcutaneous tissue, and perivascular mixed inflammatory infiltrates. These similarities support the recognition of consistent diagnostic features of PLEVA across various studies.

The differential diagnosis of PLEVA includes a wide array of inflammatory and infectious dermatoses, such as viral exanthems (e.g., varicella), Gianotti‐Crosti syndrome, small‐vessel vasculitis, lymphomatoid papulosis, and even early forms of cutaneous T‐cell lymphoma (CTCL). Immunohistochemistry and T‐cell receptor gene rearrangement studies can be helpful in challenging cases, although these resources were not accessible in our setting. PLEVA is typically a self‐limiting condition, though it may follow a relapsing–remitting course. Previous case reports have documented successful treatment with systemic corticosteroids, phototherapy, or immunosuppressants such as methotrexate. Additionally, antibiotics, particularly macrolides, have been used due to the suspected infectious or immune‐mediated pathogenesis of the disease [7, 9, 11].

In contrast, our patient achieved complete resolution with a 3‐week course of doxycycline, suggesting its potential role in the management of PLEVA. Tetracyclines like doxycycline exert not only antimicrobial effects but also significant anti‐inflammatory actions by inhibiting matrix metalloproteinases and downregulating cytokine production. This dual effect may explain their efficacy in PLEVA, even when an underlying infection is not identified [14]. Furthermore, the residual varioliform scarring and post‐inflammatory hyperpigmentation observed in this case are consistent with prior reports, reinforcing the importance of early diagnosis and treatment to minimize long‐term skin damage.

Long‐term follow‐up is essential in PLEVA, especially for monitoring recurrence, scarring, and the potential progression to lymphoproliferative disorders. Patients with atypical, persistent, or treatment‐resistant disease may benefit from periodic biopsies and molecular analyses to rule out transformation into cutaneous lymphoma [15].

## 6 Conclusion

This case underscores the rarity of mucosal involvement in PLEVA, expanding the known clinical spectrum of the disease. While our patient showed complete resolution with a 3‐week course of doxycycline, the development of varioliform scarring highlights the importance of early diagnosis and timely intervention to minimize long‐term skin sequelae. Recognizing atypical presentations is crucial for ensuring prompt treatment and preventing unnecessary complications.

## Author Contributions

**Sandesh Shah:** conceptualization, project administration, resources, writing – review and editing. **Joshana Shrestha:** conceptualization, project administration, resources, writing – original draft, writing – review and editing. **Smriti Piya:** conceptualization, project administration, writing – review and editing.

## Consent

Written patient consent has been signed and collected in accordance with the journal's patient consent policy. I will retain the original written consent form and provide it to the publisher if requested.

## Conflicts of Interest

The authors declare no conflicts of interest.

## Data Availability

Data sharing not applicable—no new data generated.
